# Rapid kill of malaria parasites by artemisinin and semi-synthetic endoperoxides involves ROS-dependent depolarization of the membrane potential

**DOI:** 10.1093/jac/dkt486

**Published:** 2013-12-12

**Authors:** Thomas Antoine, Nicholas Fisher, Richard Amewu, Paul M. O'Neill, Stephen A. Ward, Giancarlo A. Biagini

**Affiliations:** 1 Liverpool School of Tropical Medicine, Pembroke Place, Liverpool L3 5QA, UK; 2 Department of Chemistry, University of Liverpool, Liverpool L69 7ZD, UK

**Keywords:** *Plasmodium*, mitochondria, iron, haem, lipid peroxidation, free radicals, oxidative damage

## Abstract

**Objectives:**

Artemisinin and artemisinin semi-synthetic derivatives (collectively known as endoperoxides) are first-line antimalarials for the treatment of uncomplicated and severe malaria. Endoperoxides display very fast killing rates and are generally recalcitrant to parasite resistance development. These key pharmacodynamic features are a result of a complex mechanism of action, the details of which lack consensus. Here, we report on the primary physiological events leading to parasite death.

**Methods:**

Parasite mitochondrial (ΔΨ_m_) and plasma membrane (ΔΨ_p_) electrochemical potentials were measured using real-time single-cell imaging following exposure to pharmacologically relevant concentrations of endoperoxides (artemisinin, dihydroartemisinin, artesunate and the synthetic tetraoxane RKA182). In addition, mitochondrial electron transport chain components NADH:quinone oxidoreductase (alternative complex I), *bc*_1_ (complex III) and cytochrome oxidase (complex IV) were investigated to determine their functional sensitivity to the various endoperoxides.

**Results:**

Parasite exposure to endoperoxides resulted in rapid depolarization of parasite ΔΨ_m_ and ΔΨ_p._ The rate of depolarization was decreased in the presence of a reactive oxygen species (ROS) scavenger and Fe^3+^ chelators. Depolarization of ΔΨ_m_ by endoperoxides is not believed to be through the inhibition of mitochondrial electron transport chain components, owing to the lack of significant inhibition when assayed directly.

**Conclusions:**

The depolarization of ΔΨ_m_ and ΔΨ_p_ is shown to be mediated via the generation of ROS that are initiated by iron bioactivation of endoperoxides and/or catalysed by iron-dependent oxidative stress. These data are discussed in the context of current hypotheses concerning the mode of action of endoperoxides.

## Introduction

**Figure 1.**
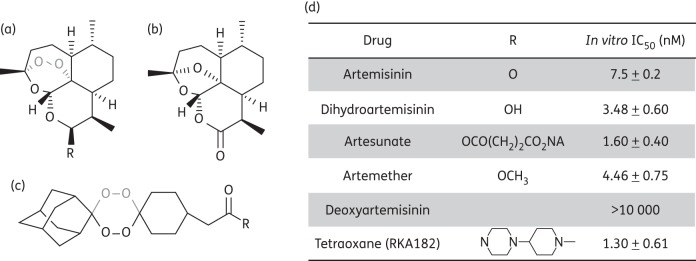
Chemical structures of endoperoxides, their corresponding *P. falciparum* growth inhibition values. Chemical structures of (a) artemisinin and its semi-synthetic derivatives, (b) deoxyartemisinin and (c) the synthetic 1,2,4,5-tetraoxane drug-development candidate RKA182 used in this study. The endoperoxide bridge is highlighted in grey. (d) *P. falciparum* growth inhibition IC_50_ values of endoperoxide antimalarials. Values are the mean ± SEM of results from three independent experiments.

Artemisinin is a tetracyclic 1,2,4-trioxane containing an endoperoxide bridge (C-O-O-C; Figure 1), the key pharmacophore of the drug.^1^ To improve the solubility and pharmacological activity of artemisinin, a first series of semi-synthetic compounds were synthesized with a similar backbone but with modifications at the C_10_ position, generating hemi-acetal, ether or ester derivatives such as dihydroartemisinin, artemether and artesunate (Figure 1). Artemisinins possess potent antimalarial activity and the WHO recommends the use of artemisinin combination therapy for first-line therapy of *Plasmodium falciparum* malaria worldwide. Based on the structure of the endoperoxide bridge, extensive studies have been devoted to the synthesis of fully synthetic endoperoxides, some of which are currently in clinical and pre-clinical development (e.g. OZ439, an ozonide designed to provide a single-dose oral antimalarial cure in humans, and the tetraoxane RKA182; Figure 1).^2,3^

The mechanism(s) of activation and subsequent biological target(s) of endoperoxides continue to be debated.^4^ The antimalarial activity of the artemisinins and related bioactive endoperoxides is believed to be mediated by activation of the endoperoxide bridge. Located in the core of the structure, its cleavage generates short-lived cytotoxic oxyradicals in the presence of haem iron or free iron Fe^2+^.^5,6^ From this premise of ‘endoperoxide bioactivation’, two different mechanisms have been proposed. The first, proposed by the Posner laboratory using ^18^O-labelled trioxane analogues, hypothesizes that the oxygen-centred radicals produced are rearranged to more stable carbon-centred radicals.^7,8^ In this ‘reductive scission’ model, ferrous iron binds to either O1 or O2 cleaving the endoperoxide bond and generating oxyradical intermediates (Figure S1, available as Supplementary data at *JAC* Online). Both radicals subsequently rearrange to primary or secondary carbon-centred radicals via either β-scission or a [1,5]-H shift. In support of this hypothesis, evidence of the formation of these carbon-centred radical intermediates has been provided using electron paramagnetic resonance spin-trapping techniques.^9–11^ It has been proposed that these C-centred radicals are capable of haem and/or protein alkylation; whilst Meunier and coworkers^12^ have provided evidence for haem alkylation, there are only a few reports of model studies on protein alkylation involving reactions with ferrous salts in the presence of cysteine (iron–sulphur chelates).^13^

In the second model, it is hypothesized that iron acts as a Lewis acid to facilitate ionic activation of antimalarial trioxanes generating downstream reactive oxygen species (ROS; Figure S1, available as Supplementary data at *JAC* Online).^14,15^ The ring opening involves heterolytic cleavage of the endoperoxide bridge followed by interaction with water generating an open, unsaturated hydroperoxide, capable of direct oxidation of protein residues. Fenton degradation of the oxygen-centred radical intermediate can provide hydroxyl radicals (HO**^·^**) highly reactive against amino acids, lipids or nucleic acids.

An alternative pathway of artemisinin bioactivation has been suggested via electron transport chain (ETC) components causing downstream ROS production and membrane depolarization in isolated *Plasmodium* mitochondria.^16,17^ Analogous to this hypothesis, a further hypothesis known as the ‘cofactor model’ proposes that endoperoxides are reduced by redox-active flavoenzymes, resulting in the perturbation of redox homeostasis coupled with the generation of ROS (Figure S1, available as Supplementary data at *JAC* Online).^18^ The cofactor model of artemisinin activation, however, is not restricted to mitochondrial flavoenzymes but rather implicates cytosolic flavoenzymes and also rejects the direct requirement for either Fe^2+^ and/or non-haem iron activation.

For models involving iron-based activation, the origin of the iron available for bioactivation is also a point of debate. There is experimental evidence for the involvement of both haem and non-haem iron in the bioactivation.^19,20^

The involvement of haem in the activation of endoperoxide compounds was first proposed following isolation of haem–artemisinin adduct in *P. falciparum* cultures.^6^ By using [^14^C]artemisinin, Maeno *et al*.^21^ showed its accumulation in the digestive food vacuole where haemoglobin degradation leads to the release of soluble haem. More recently, inhibition of haemoglobin digestion by the genetic deletion of cysteine protease falcipain-2 was shown to significantly attenuate *in vitro* endoperoxide-mediated parasite kill.^22^ Haem has been observed to enhance the oxidizing effects of endoperoxide drugs.^23^ As noted above, the reaction between haem and artemisinin has been confirmed *in vitro* and *in vivo.*^12,24,25^ In addition, it has been proposed that haem is the primary activator of artemisinin, reacting with it more efficiently than other iron-containing molecules (Fe^2+^, haemin or haemoglobin).^26^ It has also been reported that antimalarial trioxanes accumulated into the food vacuole are activated by neutral lipid-associated haem and induce oxidative membrane damage.^27,28^

However, using radio- or fluorescent-labelled artemisinins and microscopy, two studies contest the accumulation of endoperoxide drugs in the food vacuole and their possible interaction with haem.^29,30^ Additionally, Haynes *et al*.^31,32^ have proposed that artemisinins do not inhibit haemozoin formation and cannot react with haem according to conventional chemistry models. Several studies have shown that iron chelation, selective for non-haem iron, antagonizes the accumulation and antimalarial activity of endoperoxide antimalarials *in vitro* and can prevent their toxic effects in mice.^5,30,33^

Once activated, endoperoxide antimalarials have been reported to disrupt a number of parasite functions and enzymes, including the haem detoxification pathway,^23^ the translationally controlled tumour protein (*Pf*TCTP),^34^ the sarco/endoplasmic reticulum membrane calcium *Pf*ATPase6^30^ and the parasite mitochondrion. Specific targeting of parasite mitochondria by endoperoxides was reported initially based on morphological changes to mitochondria following exposure to artemether.^20,35,36^ Zhao *et al*.^37^ reported a specific inhibitory effect by artemether against *Plasmodium* cytochrome *c* oxidase (complex IV) that was later also reported by Krungkrai *et al*.^38^ based on measurements of O_2_ consumption. Li *et al*.^16^ hypothesized a role for parasite mitochondria, specifically the type II NADH:quinone oxidoreductase (*Pf*NDH2),^39,40^ by acting both as a target and as an activator of endoperoxides via electron donation by the ETC. Conflicting data have since emerged, with Crespo *et al*.^41^ only observing mitochondrial dysfunction (as determined by rhodamine 123 fluorescence) upon exposure to artemisinin after 8 h but not after 4 h, interpreting this as a downstream effect rather than an initiator of killing. Conversely, Wang *et al*.^17^ reported depolarization of the membrane potential of isolated parasite mitochondria by artemisinin following 2 h of incubation.

Studies of artemisinin toxicity in human cells indicate that respiring mitochondria play an essential role in endoperoxide-induced cytotoxicity via the generation of ROS; however, using HeLa *ρ*^0^ cells, which are devoid of a functioning ETC, it was demonstrated that the ETC does not have any role in the reductive activation of the endoperoxide to cytotoxic carbon-centred radicals.^42^

We have previously described the use of a real-time single-cell imaging method for monitoring malaria parasite mitochondrial (ΔΨ_m_) and plasma membrane potential (ΔΨ_p_)^43,44^ and reported malaria parasite ETC activities including *Pf*NDH2.^40,45^ Using these approaches, we have re-examined the effect of endoperoxides on parasite bioenergetic functions and discuss our data in the context of the current hypotheses.

## Materials and methods

### Parasites, culture and drug sensitivity testing

*P. falciparum* (3D7 strain) cultures consisted of a 2% (v/v) suspension of O+ erythrocytes in RPMI 1640 medium (R8758, glutamine and NaHCO_3_) supplemented with 10% pooled human AB+ serum, 25 mM HEPES (pH 7.4) and 20 μM gentamicin sulphate.^46^ Cultures were grown under a gaseous headspace of (in v/v) 4% O_2_ and 3% CO_2_ in N_2_ at 37°C. Parasite growth was synchronized by treatment with sorbitol.^47^ Drug susceptibilities were determined with an inoculum size of 0.5% parasitaemia (ring stage) and 1% haematocrit and were assessed by the measurement of fluorescence after the addition of SYBR Green I as described previously.^48^ Drug 50% inhibitory concentration (IC_50_) values were calculated from the log of the dose–response relationship, as fitted with Grafit software (Erithacus Software, Horley, UK). Drug stock solutions (1–10 mM) were prepared in DMSO. The results are given as the means of at least three separate experiments.

### Preparation of P. falciparum cell-free extracts

Free parasites were prepared from aliquots of infected erythrocytes (∼8 × 10^9^ cells/mL) by adding 5 volumes of 0.15% (w/v) saponin in phosphate-buffered saline (137 mM NaCl, 2.7 mM KCl, 1.76 mM K_2_HPO_4_, 8.0 mM Na_2_HPO_4_ and 5.5 mM d-glucose, pH 7.4) for 5 min, followed by three washes by centrifugation and resuspension in HEPES (25 mM)-buffered RPMI containing a protease inhibitor cocktail (Complete Mini; Roche, Mannheim, Germany). Cell extract was prepared by repeated freeze–thawing in liquid N_2_, followed by disruption with a sonicating probe.

### Preparation of decylubiquinol

The artificial quinol electron donor was prepared based on a previously described method.^49^ Briefly, 2,3-dimethoxy-5-methyl-*n*-decyl-1,4-benzoquinone (decylubiquinone), an analogue of ubiquinone (Sigma, St Louis, MO, USA), was dissolved (10 mg) in 400 μL of nitrogen-saturated hexane. An equal volume of aqueous 1.15 M sodium dithionite was added and the mixture shaken vigorously until colourless. The upper, organic phase was collected and the decylubiquinol recovered by evaporating off the hexane under N_2_. The decylubiquinol was dissolved in 100 μL of 96% ethanol (acidified with 10 mM HCl) and stored in aliquots at −80°C. Decylubiquinol concentration was determined spectrophotometrically from absolute spectra, using *ɛ*_288–320_ = 4.14 mM^−1^ cm^−1^.

### Measurement of bc_1_ protein and complex IV activities

Decylubiquinol:cytochrome *c* oxidoreductase (*bc*_1_ protein) and cytochrome *c* oxidase (complex IV) activity were assayed in a Cary 4000 spectrophotometer (Varian Inc., USA). The *bc*_1_ reaction buffer consisted of 50 mM potassium phosphate (pH 7.5), 2 mM EDTA, 10 mM KCN (1 M stock solution, pH adjusted to 7.5) and 30 μM horse heart cytochrome *c* (oxidized) (Sigma).^49^ The complex IV mixture is composed of 50 mM potassium phosphate (pH 7.5), 2 mM EDTA and 5 μM antimycin. Inhibitors were added prior to the addition of substrate. The reaction volume was 700 μL and assays were performed at room temperature. *P. falciparum bc*_1_ and complex IV were assayed from cell-free *P. falciparum* extract at a total protein concentration of 30–60 μg/mL. Cytochrome *c* reductase (*bc*_1_) and oxidase (complex IV) activities were initiated, respectively, by the addition of 50 μM decylubiquinol (dQH_2_) and 30 μM equine cytochrome *c* (reduced). The horse heart cytochrome *c* was reduced by sodium dithionite and then passed through a PD-10 desalting column (Pharmacia, Piscataway, NJ, USA). Activities were measured by monitoring the cytochrome *c* (reduced) concentration at 550–542 nm (*ɛ*_550–542_ = 18.1 mM^−1^ cm^−1^).

### Measurement of PfNDH2 activity

*Pf*NDH2 enzyme activity was determined based on a modification of the NADH:quinone oxidoreductase assay previously described.^50^ Enzyme activity was measured in a buffered solution (final volume 0.7 mL) containing 50 mM potassium phosphate (pH 7.5), 2 mM EDTA, 10 mM KCN and 50 μM coenzyme Q_1_ at room temperature. Recombinant *Pf*NDH2 enzyme was added as an *Escherichia coli* crude membrane preparation at a total protein concentration of between 10 and 20 μg/mL.^40^ Inhibitors were added before initiation of the reaction by addition of 200 μM NADH. *Pf*NDH2 activity was measured spectrophotometrically by monitoring the decrease of NADH concentration at 340 nm (*ɛ*_340_ = 6.22 mM^−1^ cm^−1^) and Q_1_ concentration at 283 nm (*ɛ*_283_ = 8.1 mM^−1^ cm^−1^).

### Real-time single-cell monitoring of membrane potential

The rhodamine derivative tetramethyl rhodamine ethyl ester (TMRE) was used to monitor the membrane potential of the cytoplasm and mitochondria from malaria-infected red blood cells. TMRE is cationic and reversibly accumulates inside energized membranes according to the Nernst equation. For the experiment, suspensions (1%) of infected erythrocytes in HEPES-buffered RPMI medium (no serum) were loaded with 250 nM TMRE (Molecular Probes, Eugene, OR, USA) for 10 min at 37°C. For imaging, malaria parasite-infected erythrocytes were immobilized using polylysine-coated coverslips in a Bioptechs FCS2 perfusion chamber (Bioptechs, Butler, PA, USA) and maintained at 37°C in growth medium (no serum). Inhibitors were added to the perfusate and the membrane potential-dependent fluorescence responses were monitored in real time. During all manipulations, the concentration of TMRE in the perfusate was kept at 250 nM. The fluorescence signals from malaria-infected erythrocytes were collected on a Zeiss Pascal confocal laser scanning microscope through a Plan-Apochromat 63× 1.2 numerical aperture water objective. Excitation of TMRE was performed using the HeNe laser line (543 nm). Emitted light was collected through a 560 nm long-pass filter from a 543 nm dichroic mirror. Photobleaching (the irreversible damage of TMRE producing a less fluorescent species) was assessed by continuous exposure (5 min) of loaded cells to laser illumination. For each experiment, the laser illumination and microscope settings that gave minimal reduction in signal were used. Data capture and extraction were carried out with Zeiss Pascal software and plot design was performed with Kaleidagraph (Synergy Software, Reading, PA, USA).

## Results

### Endoperoxides have a minimal inhibitory effect on the major mitochondrial respiratory chain components

As described, there is conflicting evidence on the role of the parasite ETC in the activation and/or resultant mitochondrial dysfunction following the exposure of parasites to endoperoxides. To determine whether there is a direct inhibitory effect of endoperoxides on ETC components, activities from three of the main ETC enzymes, *Pf*NDH2, *bc*_1_ complex and cytochrome *c* oxidase, were measured directly in the presence of a number of endoperoxides. As described in the Materials and methods section, the *bc*_1_ complex (complex III) and cytochrome *c* oxidase (complex IV) were measured directly from parasite cell-free extracts, whilst *Pf*NDH2 activity was measured from membrane preparations of a heterologous expression system described previously.^40^ No or relatively weak (∼20%) inhibition of the individual respiratory components was observed for all the endoperoxides tested at comparatively high fixed doses (1 μM final concentration; Table 1). Positive controls using selective inhibitors of the individual respiratory components were consistent with previously reported inhibitory values (Table 1).

**Table 1.** Inhibitory profiles of endoperoxide compounds for three major components of the ETC of *P. falciparum* (3D7 strain)

Inhibitor	Percentage inhibition		
	*Pf*NDH2	*bc*_1_ (complex III)	complex IV
Artemisinin (1 μM)	17.1 ± 4.2	3.2 ± 3.7	18.4 ± 4.3
Tetraoxane (1 μM)	12.3 ± 4.9	23.2 ± 4.2	19.1 ± 2.0
Dihydroartemisinin (1 μM)	5.0 ± 4.3	6.7 ± 4.1	21.1 ± 7.9
Artesunate (1 μM)	3.7 ± 2.9	2.3 ± 0.4	21.1 ± 3.9
Artemether (1 μM)	4.1 ± 3.3	1.8 ± 1.9	23.0 ± 2.0
HDQ (100 nM)	88.1 ± 0.5	ND	ND
Atovaquone (50 nM)	ND	89.0 ± 1.0	ND
Cyanide (15 mM)	ND	ND	100 ± 0.0

Specific inhibitors of *Pf*NDH2 [1-hydroxy-2-dodecyl-4(1H)quinolone; HDQ], *bc*_1_ protein (atovaquone) and complex IV (cyanide) were used as positive controls. Direct activity assays were performed on recombinant *Pf*NDH2 enzymes, whereas *bc*_1_ protein and complex IV were assayed with cell-free parasite extracts.

Inhibitor concentrations used are indicated in brackets.

Values are means ± SEM; *n* = 3 independent experiments. ND, not determined.

### Endoperoxides collapse membrane potential-dependent accumulation of TMRE in P. falciparum-infected erythrocytes

To determine the effect of endoperoxides on membrane potential, a real-time single-cell imaging approach was used. The measurement is based on the accumulation of the cationic fluorescence probe TMRE according to the Nernst equation. Due to dynamic fluorescence measurements, the probe is subject to photobleaching. To minimize this, several parameters of the confocal laser scanning microscope (laser power, scan speed, pinhole diameter, number of scan sweeps and degree of magnification) were optimized before recording each experiment. Upon addition of TMRE to *P. falciparum*-infected erythrocytes, a strong fluorescence signal was observed from the whole cytosol (except the food vacuole), corresponding to the addition of plasma and mitochondrial membrane potential. For all assays, the fluorescence dynamic range was set up so that untreated TMRE-loaded cells were regarded as having complete fluorescence (100%), whereas the baseline (0%) was set by the addition of 10 μM H^+^ ionophore carbonyl cyanide *p*-(trifluoromethyl)phenylhydrazone (FCCP).

**Figure 2.**
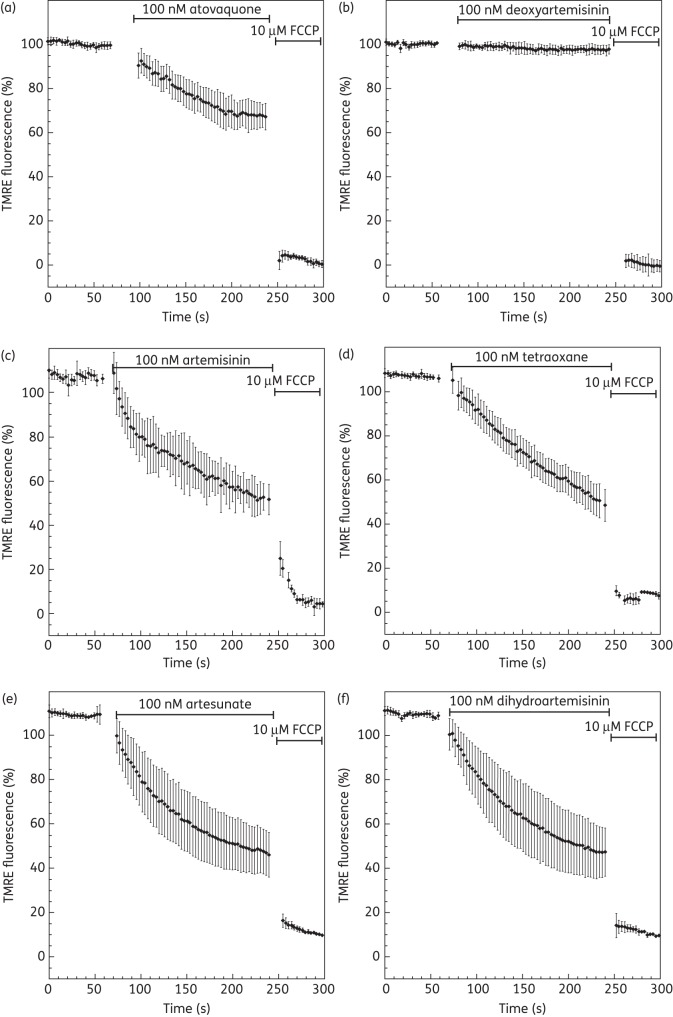
Effect of endoperoxide antimalarials on both the plasma and mitochondrial membrane potential of *P. falciparum*. Time course of TMRE-dependent fluorescence of *P. falciparum*-infected erythrocytes after the addition of (a) 100 nM atovaquone, (b) 100 nM deoxyartemisinin, (c) 100 nM artemisinin, (d) 100 nM tetraoxane (compound RKA182), (e) 100 nM artesunate and (f) 100 nM dihydroartemisinin. The data were normalized to 100% in untreated cells and to 0% in FCCP (10 μM)-treated cells. Graphs show means from experiments performed independently ± standard errors (*n *≥ 7).

The endoperoxide compounds used in this study were shown to inhibit parasite growth in the low nanomolar range, consistent with previous studies (Figure 1). Addition of endoperoxides to trophozoite-stage parasites (100 nM) resulted in a 55%–60% reduction of total membrane potential-dependent fluorescence within <3 min (Figure 2c–f). Using the same conditions, atovaquone, the selective *bc*_1_ complex inhibitor, decreased the total membrane potential-dependent fluorescence by 30% (Figure 2a), consistent with previous observations of the mitochondrial contribution to fluorescence.^43^ In contrast, addition of deoxyartemisinin, which lacks the endoperoxide bridge (Figure 1), resulted in the minimal loss of membrane potential-dependent fluorescence, consistent with its poor antimalarial activity (Figure 2b).

Although these experiments demonstrate for the first time the rapid depolarization of membrane potential upon exposure to endoperoxides, since there is a contribution from both plasma (ΔΨ_p_) and mitochondrial membrane (ΔΨ_m_) potentials to TMRE accumulation, further experiments were performed to measure ΔΨ_p_ and ΔΨ_m_ independently.

### Depolarization of the mitochondrial membrane potential by endoperoxides

**Figure 3.**
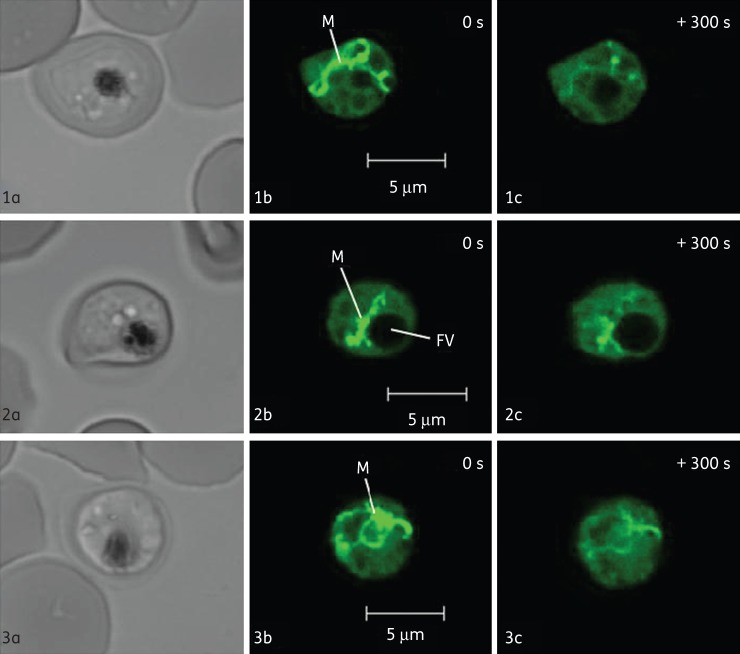
Effect of atovaquone, artemisinin and tetraoxane (RKA182) on fluorescent mitochondria from *P. falciparum* trophozoites. Bright-field fluorescence (a) and TMRE fluorescence images of concanamycin-pretreated infected erythrocytes before induction (b) and 300 s after induction (c) with 100 nM atovaquone (1), 100 nM artemisinin (2) and 100 nM tetraoxane (3). The green in these images is a pseudocolour. ‘M’ indicates the parasite mitochondrion and ‘FV’ indicates the food vacuole.

**Figure 4.**
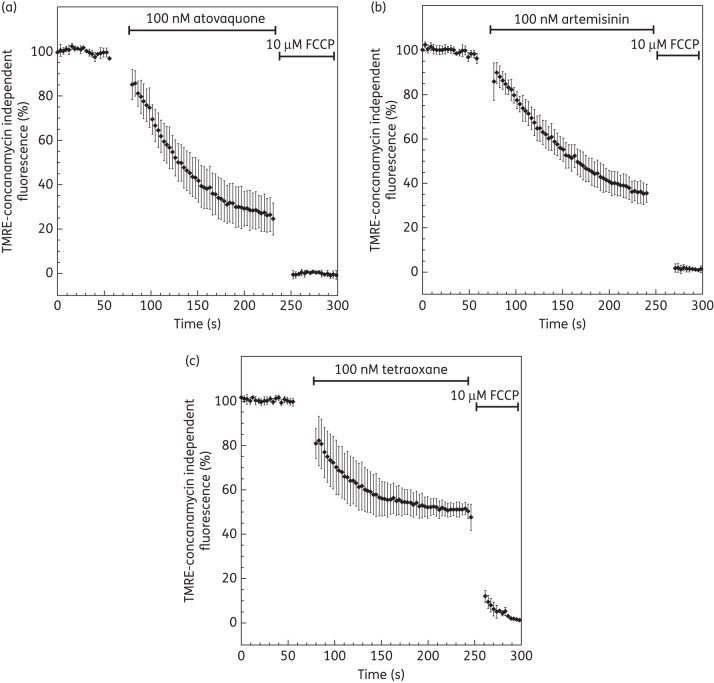
Effect of artemisinin and tetraoxane on mitochondrial membrane potential only. Plasma membrane potential is depolarized by treatment of *P. falciparum*-infected erythrocytes with 200 nM concanamycin A before addition of inhibitors. Time course of TMRE-plasma independent fluorescence is followed after addition of (a) 10 nM atovaquone, (b) 100 nM artemisinin and (c) 100 nM tetraoxane. Data were normalized to 100% in untreated cells and to 0% in FCCP (10 μM)-treated cells. Graphs show means from experiments performed independently ± standard errors (*n* ≥ 7).

To evaluate the impact of endoperoxides on ΔΨ_m_, cells were pretreated with concanamycin A, a V-type H^+^-ATPase inhibitor. Upon addition of concanamycin A (200 nM), the fluorescence intensity from the cytosol decreases ∼70%–80%,^43^ leaving a local and strong signal originating from the parasite mitochondrion, as demonstrated in Figure 3(b). To measure ΔΨ_m_-dependent fluorescence, concanamycin A-treated parasites were normalized to 100% and the baseline (0%) was set by FCCP addition (10 μM). Atovaquone addition rapidly (≈3 min) reduced concanamycin-independent TMRE fluorescence by 70% (Figures 3 and 4a). Similarly, both artemisinin and tetraoxane decreased the concanamycin-independent TMRE fluorescence by 60% and 50%, respectively (Figures 3 and 4b and c).

### Depolarization of the plasma membrane potential by endoperoxides

**Figure 5.**
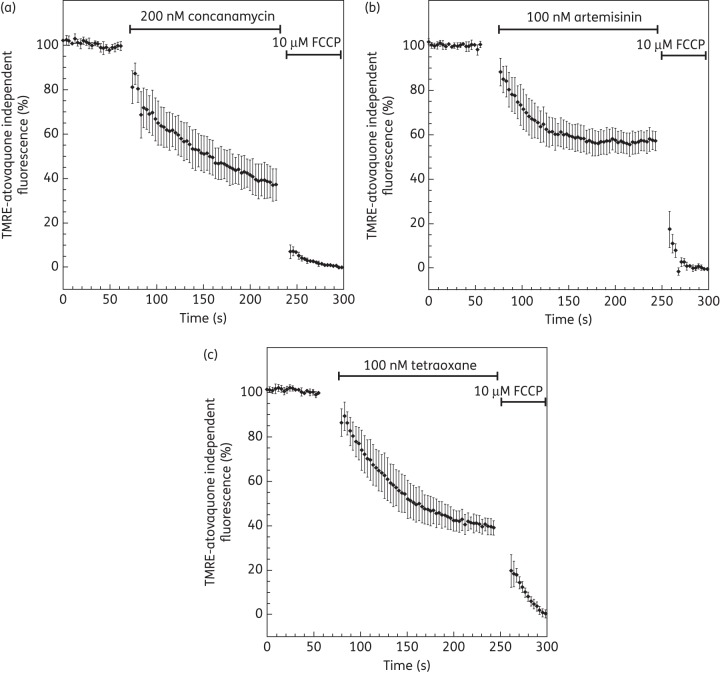
Effect of artemisinin and tetraoxane on plasma membrane potential only. Mitochondrial membrane potential is depolarized by treatment of *P. falciparum*-infected erythrocytes with 100 nM atovaquone before addition of inhibitors. Time course of TMRE-mitochondrial independent fluorescence is followed after addition of (a) 200 nM concanamycin A, (b) 100 nM artemisinin and (c) 100 nM tetraoxane. Data were normalized to 100% in untreated cells and to 0% in FCCP (10 μM)-treated cells. Graphs show means from experiments performed independently ± standard errors (*n* ≥ 7).

The ΔΨ_m_ was demonstrated to represent between 20% and 30% of the total cellular TMRE fluorescence, leaving a homogeneous cytosolic signal originating from the plasma membrane only.^43^ To evaluate the ΔΨ_p_-dependent fluorescence, parasites were pretreated with 100 nM atovaquone for 5 min before addition of the endoperoxide inhibitor. For these experiments, atovaquone-treated parasites were normalized to 100% and the baseline (0%) was set by FCCP addition (10 μM). The vacuolar H^+^-ATPase is involved in transforming the energy of ATP hydrolysis to generate the electrochemical potential at the surface of the malaria parasite through the transport of H^+^ across the plasma membrane.^51^ As expected, the addition of concanamycin A, a well-known V-type ATPase inhibitor, rapidly decreased ΔΨ_p_-dependent fluorescence by 60% (Figure 5a). In a similar manner, artemisinin and tetraoxane were observed to decrease the atovaquone-independent fluorescence signal by 40% and 60%, respectively (Figure 5b and c).

The described data confirm that both ΔΨ_m_ and ΔΨ_p_ are rapidly depolarized upon exposure to physiological concentrations of endoperoxides. The next set of experiments was performed to ascertain whether the rapid depolarization would be affected by chelation of free Fe^3+^, as previously hypothesized by other studies.

### Effect of Fe^3^^+^ chelators desferrioxamine (DFO) and deferipone (DFP) on the membrane potential depolarization by artemisinin and tetraoxane

DFO and DFP, two chelating agents selective for non-haem Fe^3+^,^52^ were used at a fixed dose to determine the effect of free iron on the rapid endoperoxide-induced, parasite total (ΔΨ_m_ and ΔΨ_p_) membrane potential depolarization.

**Figure 6.**
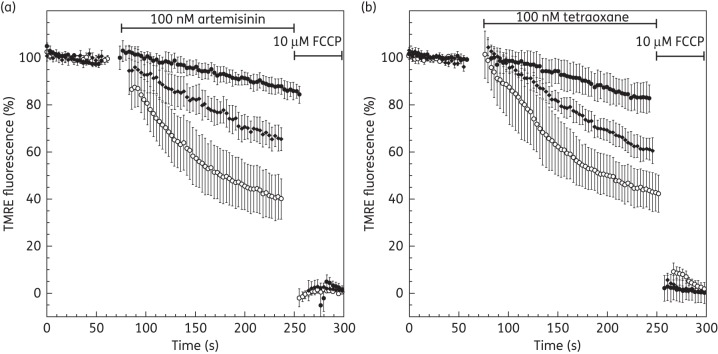
Effect of artemisinin and tetraoxane on the membrane potential in the presence of iron chelators. Time course of TMRE-dependent fluorescence of *P. falciparum*-infected erythrocytes after addition of (a) 100 nM artemisinin and (b) 100 nM tetraoxane. Cells are not treated (open circles) or subjected to iron chelator treatment with 100 μM DFO (filled circles) or 100 μM DFP (filled diamonds). Data were normalized to 100% in untreated cells and to 0% in FCCP (10 μM)-treated cells. Graphs show means from experiments performed independently ± standard errors (*n* ≥ 7).

Growth inhibition studies (48 h IC_50_) using both iron chelators revealed moderate antimalarial activity against 3D7 *P. falciparum* of 17.3 ± 2 μM (DFO) and 111.8 ± 2 μM (DFP). However, during the short time period of the single-cell assays (<6 min), the addition of 100 μM DFO or 100 μM DFP to the perfusate did not result in a decrease of parasite ΔΨ_m_ and ΔΨ_p_-dependent fluorescence signal. However, addition of DFO and DFP (100 μM) to the perfusate was observed to significantly minimize the depolarization caused by either artemisinin (100 nM; Figure 6a) or tetraoxane (100 nM; Figure 6b), relative to chelator-free controls. For both endoperoxides, DFO was observed to confer greater protection than DFP.

Next, it was investigated whether ROS scavengers would also confer a protective effect against endoperoxides as postulated in previous studies.

### Effect of the superoxide scavenger Tiron on the membrane potential depolarization induced by artemisinin and tetraoxane

**Figure 7.**
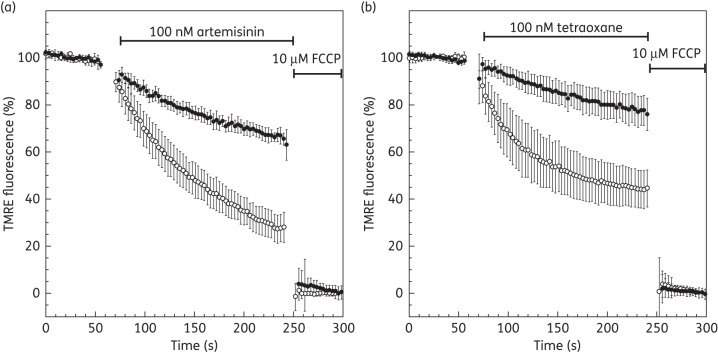
Effect of artemisinin and tetraoxane on the membrane potential in the presence of ROS scavenger. Time course of TMRE-dependent fluorescence of *P. falciparum*-infected erythrocytes after addition of (a) 100 nM artemisinin and (b) 100 nM tetraoxane. Cells are not treated (open circles) or subjected to ROS scavenger treatment with 100 μM Tiron (filled circles). Data were normalized to 100% in untreated cells and to 0% in FCCP (10 μM)-treated cells. Graphs show means from experiments performed independently ± standard errors (*n* ≥ 7).

Tiron, a cell membrane-permeable superoxide scavenger, was added (100 μM) to the perfusate and endoperoxide-induced ΔΨ_m_ and ΔΨ_p_ depolarization was measured as described previously. Tiron was clearly observed to have a significant protective effect against endoperoxide-induced membrane potential depolarization, decreasing the rate of depolarization by >50% for both artemisinin and tetraoxane (Figure 7). In control experiments, Tiron alone had no effect on parasite ΔΨ_m_ and ΔΨ_p_ based on TMRE fluorescence (data not shown).

## Discussion

### ETC components are not a direct target for endoperoxides

Specific inhibition by endoperoxides has previously been reported for *Plasmodium* cytochrome *c* oxidase (complex IV)^37,38^ and type II NADH:quinone oxidoreductase (*Pf*NDH2).^16^ In agreement with previous studies, we also noted inhibition of complex IV but only to a maximum of 20% at relatively high concentrations (1 μM) of a range of endoperoxides (Table 1). Little or no inhibition was observed, however, for either recombinant *Pf*NDH2 or *bc*_1_ (Table 1). It should be noted that all enzymatic assays were performed using either cell-free extracts or *E. coli* membrane preparations and would be expected to have contained trace Fe^2+^ and, for the parasite extract, haem. These data indicate that ETC components are not direct targets for endoperoxide inhibition in *Plasmodium*. Previous studies reporting inhibition of *Pf*NDH2 by endoperoxides^16^ did not measure the enzyme directly, but rather showed increased sensitivity to endoperoxides in yeast transfected with *Pf*NDH2. It is likely, therefore, that *Pf*NDH2 in the yeast system increased sensitivity to endoperoxides via an indirect effect, possibly by serving as a source of ROS by electron transfer from the *Pf*NDH2 flavin to O_2_ or potentially directly to the endoperoxides.

### Rapid loss of parasite vitality upon exposure to endoperoxides

In unicellular organisms, life can be defined as the maintenance of an electrochemical potential across the organism's outer membrane (negative entropy).^53^ Plasmodia have a proton potential across the plasma membrane (ΔΨ_p_) of ∼−95 mV.^51,54^ It is generated by V-type H^+^-ATPases, which transfer protons out of the parasite cytosol.^55,56^ Localized in the plasma membrane, the V-type H^+^-ATPase also plays a role in regulating a neutral cytosolic pH (near 7.3), crucial for enzyme function, incorporation of vitamin B5 and generation of a pH gradient across membranes of internal organelles.^56,57^ The maintenance of a ΔΨ_p_ is also important in mediating the influx of K^+^ in the parasite cytosol and the uptake of nutrients such as choline.^51,58,59^ The direct inhibition of the V-type H^+^-ATPase by specific inhibitors such as bafilomycin A_1_ or concanamycin A has been demonstrated to depolarize the plasma membrane potential and disrupt the physiology of the parasite, leading to its death.^43,51,57^ The parasite mitochondrial membrane potential (ΔΨ_m_) is generated via the ETC, through the activity of the *bc*_1_ complex and cytochrome *c* oxidase.^43,60^ An essential function of the mitochondrion for parasite survival during the intraerythrocytic stages is the provision of orotate for pyrimidine biosynthesis through the activity of dihydroorotate dehydrogenase.^61,62^

Exposure of *P. falciparum*-infected erythrocytes to pharmacologically relevant concentrations of endoperoxides resulted in a rapid loss of membrane potential-dependent accumulation of TMRE (Figure 2). On account of the comparatively high contribution by the ΔΨ_p_ (relative to mammalian cells), it was not possible from these experiments to distinguish whether the depolarization was on account of an effect on the ΔΨ_p_ alone and/or the ΔΨ_m_. Inhibition of the parasite V-type H^+^-ATPase by addition of concanamycin A to the perfusate ‘unmasked’ the parasite to reveal the ΔΨ_m_-dependent accumulation of TMRE (Figure 3). With this manipulation, it was possible to determine that for all the endoperoxide classes tested, ΔΨ_m_ was rapidly depolarized (Figure 4). These data are consistent with the findings by Wang *et al*.,^17^ who reported depolarization of the membrane potential of isolated parasite mitochondria by artemisinin following 2 h of incubation. Crespo *et al*.^41^ reported mitochondrial dysfunction following exposure to artemisinin only after 8 h but not after 4 h, interpreting this as a downstream effect. We note, however, that the Crespo *et al*. study did not distinguish between ΔΨ_p_- or ΔΨ_m_-dependent rhodamine accumulation and that a washing step occurred in between rhodamine staining and microscopy. As rhodamine is a cationic fluorophore, there is a balance between the concentration gradient of the probe and the total ΔΨ according to the Nernst equation: *Ψ* = *RT*/*F* ln(*P*_in_/*P*_out_), where *R*, *T*, *F*, *D*_in_ and *D*_out_ represent the universal gas constant, the absolute temperature, the Faraday constant and the intra- and extracellular probe concentrations, respectively. The washing step would therefore affect the distribution of the probe in a time-dependent manner and may account for the discord between the data of Crespo *et al*.^41^ and those presented here and by Wang *et al*.^17^

Pretreatment of parasites with atovaquone in the perfusate further allowed the measurement of ΔΨ_p_ alone_._ Exposure to endoperoxides resulted in the rapid depolarization of parasite ΔΨ_p_ (Figure 5). The rapid onset of ΔΨ_p_ depolarization exposed to pharmacologically relevant concentrations of endoperoxides indicates that this is a primary pharmacodynamic event leading to parasite death and is consistent with *in vitro*^63^ and *in vivo*^64,65^ studies reporting the rapid killing rate of the endoperoxide class. The rapid onset of ΔΨ_p_ depolarization by endoperoxides is also consistent with studies demonstrating that short pulses of artemisinins (1–6 h) are sufficient for parasite kill, albeit with stage-dependent differences.^66,67^ It is also noteworthy that disruption of the parasite transmembrane pH gradient via inhibition of the V-type ATPase has been reported to drop the cytosolic pH by 0.4 pH units in <3 min and 0.5–0.6 units within 20 min,^56,57^ leading to the inhibition of parasite growth within 30 min to 4 h (depending on inhibitor concentration).^57^

Deoxyartemisinin did not to have any depolarizing effect on either parasite ΔΨ_p_ or ΔΨ_m_ (Figure 2). This result is in line with previous studies^17,68,69^ and confirms the importance of the endoperoxide bond in mediating antimalarial activity.

### Rapid depolarization of the parasite ΔΨ_p_ involves iron and ROS

The parasite's unique ability to digest haemoglobin is generally accepted to confer selective toxicity to endoperoxides (e.g. Klonis *et al*.^22^) either directly, by activation of the endoperoxides by Fe^2+5^ and/or haem,^70^ or indirectly through the ability of Fe^3+^ to oxidize cytosolic (reduced) cofactors.^18^ In this study, we used DFO and DFP, two known chelators of Fe^3+^,^71^ to determine the effect on endoperoxide-induced ΔΨ_p_ depolarization. Addition of the chelators to the perfusate was clearly observed to confer a protective effect to the parasite upon exposure to the endoperoxides (Figure 6). These data can be interpreted to suggest that Fe^3+^ is involved in the depolarization of the parasite membrane. The iron-mediated hypothesis of endoperoxide activation identifies a role for Fe^2+^. As the chelators have a much higher affinity for Fe^3+^ compared with Fe^2+^, such a mechanism would necessitate the additional presence of redox cycling activities, such as thiols GSH ↔ GSSG (reduced and oxidized glutathione, respectively) and/or flavin enzymes. In previous experiments performed *in situ* using single-cell imaging, we observed that endoperoxide–acridine adducts could be completely washed out in the presence of DFO, but remained irreversibly bound in the absence of DFO, suggesting that the drugs were being activated by iron to form stable covalent adducts.^52^ It is therefore possible that the protective effect of the iron chelators observed in our studies here is a result of a decrease in the bioactivation of the endoperoxides. An alternative or additional explanation is that the iron chelators conferred a protective effect by decreasing the generation of ROS through Fe^2+^-mediated oxidative stress such as the Fenton reaction, generating the highly membrane damaging hydroxyl radical (**^·^**OH). It is also noteworthy that DFO has been reported to be able to directly scavenge ROS.^72^ It should also be noted that in the experiments presented here, intracellular haem concentrations cannot be selectively and rapidly manipulated; therefore, the potential role of haem in the depolarization of ΔΨ_p_ by endoperoxides cannot be elucidated.

Although the exact protective mechanism for iron cannot be deduced from our data, a role for ROS is clearly demonstrated in experiments using Tiron, the superoxide anion (**^·^**O_2_^−^) scavenger, showing a decreased rate of membrane depolarization following exposure to endoperoxides (Figure 7). The indiscriminate nature of the depolarization of both ΔΨ_p_ or ΔΨ_m_ is suggestive of a mechanism involving the general lipid peroxidation of the parasite membranes; however, in addition to this, a specific inhibition of key enzymes such as the V-type ATPase by ROS cannot be ruled out.^73^

### Conclusions

In summary, this study reports that rapid loss of ΔΨ_p_ following exposure to endoperoxides is a primary physiological event leading to parasite death. A loss of ΔΨ_m_ is also reported, but the lack of direct inhibition of ETC H^+^-pumping complexes by endoperoxides and the indiscriminate nature of membrane depolarization is consistent with a non-ETC mode of action, as reported recently in mammalian cells.^42^ The rapid depolarization of ΔΨ_p_ by endoperoxides involves ROS and iron, but it is not discernible from this study whether iron plays a role in endoperoxide bioactivation and/or iron-mediated oxidative stress.

## Funding

This work was supported by grants from the MRC, Leverhulme Trust, the Wellcome Trust and the European Union Framework 7 Marie Curie Initial Training Network 215281 (InterMalTraining).

## Transparency declarations

None to declare.

## Supplementary data

Figure S1 is available as Supplementary data at *JAC* Online (http://jac.oxfordjournals.org/).

Supplementary Data
