# Supplementary material for: Rapid kill of malaria parasites by artemisinin and semi-synthetic endoperoxides involves ROS-dependent depolarization of the membrane potential
Source: J Antimicrob Chemother. 2013 Dec 12;69(4):1005–16. doi: 10.1093/jac/dkt486 (PMC3956377; doi:10.1093/jac/dkt486)
Supplement: Supplementary Data [file supp_69_4_1005__index.html]

Rapid kill of malaria parasites by artemisinin and semi-synthetic endoperoxides involves ROS-dependent depolarization of the membrane potential — Rapid kill of malaria parasites by artemisinin and semi-synthetic endoperoxides involves ROS-dependent depolarization of the membrane potential — Supplementary Data 

# Rapid kill of malaria parasites by artemisinin and semi-synthetic endoperoxides involves ROS-dependent depolarization of the membrane potential

## Supplementary Data

Supplementary Data

**Files in this Data Supplement:**

- Supplementary Data - Docx file
