# Supplementary material for: Rapid kill of malaria parasites by artemisinin and semi-synthetic endoperoxides involves ROS-dependent depolarization of the membrane potential
Source: J Antimicrob Chemother. 2013 Dec 12;69(4):1005–16. doi: 10.1093/jac/dkt486 (PMC3956377; doi:10.1093/jac/dkt486)
Supplement: Supplementary Data [file supp_dkt486_dkt486supp.docx]

**Supplementary data**

**Figure S1.** (a) Reductive scission models through iron-activation pathways resulting in the formation of oxy-radicals or carbon-centred radicals. (b) Cofactor model (R=cofactor residue) – where artemisinin is not required to be directly activated by iron and functions as a cofactor oxidant.

a

b
